# Geographical Differences and Their Associated Factors in Chronic Obstructive Pulmonary Disease Mortality in Japan: An Ecological Study Using Nationwide Data

**DOI:** 10.3390/ijerph182413393

**Published:** 2021-12-20

**Authors:** Tasuku Okui, Jinsang Park

**Affiliations:** 1 Medical Information Center, Kyushu University Hospital, Fukuoka 812-8582, Japan; 2 Department of Pharmaceutical Sciences, International University of Health and Welfare, Fukuoka 831-8501, Japan; park21@iuhw.ac.jp

**Keywords:** vital statistics, Japan, mortality, municipalities, chronic obstructive pulmonary disease

## Abstract

Geographical differences in chronic obstructive pulmonary disease (COPD) mortality have not been determined using municipal-specific data in Japan. This study determined the geographical differences in COPD mortality in Japan using municipal-specific data and identified associated factors. Data on COPD mortality from 2013 to 2017 for each municipality were obtained from the Vital Statistics of Japan. We calculated the standardized mortality ratio (SMR) of COPD by an empirical Bayes method for each municipality and located the SMRs on a map of Japan. In addition, an ecological study was conducted to identify factors associated with the SMR using demographic, socioeconomic, and medical characteristics of municipalities by a spatial statistics model. Geographical differences in the SMR were different in men and women, and municipalities with a low SMR tended to be more frequent in women. Spatial regression analysis identified that the total population and taxable income per capita were negatively associated with the SMR in men. In women, population density, the proportion of fatherless households, and the number of clinics per capita were positively associated with the SMR, whereas taxable income per capita was negatively associated with the SMR. There were some differences in regional characteristics associated with COPD mortality by sex.

## 1. Introduction

Chronic obstructive pulmonary disease (COPD) is a leading cause of death, and more than 3.2 million people died from the disease worldwide in 2015 [1]. The COPD mortality rate in Japan is relatively low [2], but more than 15,000 people die each year [3]. In addition, COPD prevalence in Japan is not different from other countries [4], and the economic cost associated with COPD is high due to circumstances such as inpatient hospitalization costs or work disability [5,6]. COPD exacerbations can cause severe dyspnea [7], impairing patients’ health-related quality of life [8]. Further, COPD is considered a preventable chronic lung disease, as long-term exposure to cigarette smoke is the major cause of COPD in Japan [2,4].

In Japan, clinical risk factors associated with COPD prevalence or mortality have been investigated [9,10,11]; however, studies of the association between socioeconomic status or patients’ place of residence and COPD prevalence or mortality are few [12,13]. The association between COPD mortality and socioeconomic status has been reported in other countries [14,15]. In contrast, no study has investigated the socioeconomic and demographic characteristics associated with COPD mortality using nationwide statistical data in Japan. One common method for investigating attributes related to disease mortality is an ecological study. This study can identify factors associated with regional differences in disease mortality.

Geographical differences in COPD incidence, mortality, and prevalence have been reported in other countries [16,17,18,19,20,21], and factors associated with these regional differences have been identified. A study in Taiwan has reported that factors such as smoking prevalence, percentage of aborigines, and concentration of particulate matter 10 are positively associated with regional COPD mortality rate [20], and another study in China has showed that smoking and exposure to particulate matter 2.5 are positively associated with COPD prevalence [18]. Another study that investigated factors associated with COPD mortality in Shanghai has revealed that extremely low temperatures in the month before death, shorter distances to the highway, and lower gross domestic product are associated with mortality [21]. Therefore, socioeconomic and environmental characteristics are particularly associated with regional COPD mortality or prevalence in other countries.

In contrast, in Japan, prefecture-specific data have been used to identify regional differences in COPD mortality [22], but geographical differences using municipality-specific data have not been identified. We can infer demographic, socioeconomic, and medical characteristics associated with COPD mortality by identifying geographical differences and associated factors using municipality-specific data. This study identified geographical differences and associated factors in COPD mortality in Japan using municipal-specific data.

## 2. Materials and Methods

COPD mortality data from 2013 to 2017 for each municipality and COPD mortality data from 2013 to 2017 by sex and age in all of Japan were obtained from the Vital Statistics of Japan [3]. The Vital Statistics of Japan covers all mortality in Japan. The International Statistical Classification of Diseases and Related Health Problems 10th edition code corresponding to COPD mortality in the Vital Statistics is J41–J44. We used mortality data from 2013 to 2017 because the census was conducted in 2015, and data on municipal characteristics can be obtained in these periods. Population data of each municipality by sex and age from 2013 to 2017 were obtained from the “Survey on population, demographics, and number of households based on the Basic Resident Register” [23].

We used demographic, socioeconomic, and medical characteristics of municipalities that can be obtained from government statistics as explanatory variables for identifying factors associated with COPD mortality. Specifically, we used total population, population density, the proportion of single households, the proportion of fatherless households, the proportion of divorced individuals, the proportion of unemployed individuals, the proportion of blue-collar workers, taxable income per capita, the number of hospital beds per 100,000 persons, the number of clinics per 100,000 persons, the number of hospitals per 100,000 persons, and the number of physicians per 100,000 persons in municipalities as explanatory variables. Data on the municipal area were obtained from the survey on nationwide municipal areas by the Ministry of Land, Infrastructure, Transport and Tourism [24]. The number of single households, fatherless households, total households, divorced individuals, unemployed individuals, blue-collar workers, and the labor force individuals were obtained from the census [24,25]. The labor force individuals include employed and unemployed individuals. Proportions for the household-related variables were calculated by dividing the corresponding numbers by the number of total households. Proportions for the variables related to workers were calculated by dividing the corresponding numbers by the number of labor force persons. Taxable income data were obtained from the “Survey on taxation status of municipal taxes” [24]. Data on the number of hospital beds, number of clinics, number of hospitals, and number of physicians were obtained from the Survey on Medical Institutions and the Statistics of Physicians, Dentists, and Pharmacists [24]. All data of explanatory variables in 2015 were used in the analysis except for the number of physicians because it was not surveyed in 2015; the number of physicians in 2014 was used in the analysis. In addition, map data of municipalities in Japan were obtained from the digital national land information of the Ministry of Land, Infrastructure, Transport and Tourism [26].

Data from all municipalities in Japan were used in this study. There are 47 prefectures in Japan, and these prefectures consist of 1741 municipalities in total. A municipality is the smallest unit of a region described in the Vital Statistics of Japan.

For statistical analyses, firstly, the COPD mortality rate by sex and age group was calculated using the data on COPD mortality and population of Japan. By multiplying the COPD mortality rate with the population by age group for each sex and municipality, we calculated the expected COPD mortality for each age group, sex, and municipality. By summing the expected mortality of all the age groups, we calculated the expected COPD mortality for each sex and municipality. The standardized mortality ratio (SMR) of COPD can be calculated for each sex and municipality by dividing the actual COPD mortality by the expected COPD mortality. In this study, an empirical Bayes method by DCluster (https://cran.r-project.org/web/packages/DCluster/DCluster.pdf (accessed on 30 November 2021)) [27] was used for calculating the SMR. Using this method, we can estimate the SMR of municipalities with zero COPD mortality owing to the low population. We mapped the SMRs on a map of Japan and identified geographical differences in COPD mortality.

We identified predictors of COPD mortality by regression analysis, and the SMR was used as the outcome variable. As it is considered that there is a spatial correlation in the SMR among municipalities, we used a spatial model for our analysis. A spatial regression model is one that is often used when analyzing spatial data, wherein a spatial correlation generally exists among the observations, such as regional data. An assumption of the independence of observations in an ordinary linear regression model is generally incorrect. In this case, using an ordinary linear regression model can lead to a biased estimate. By using a spatial model, we can estimate the coefficient of each explanatory variable, taking into account the correlation. We created a spatial matrix among municipalities based on whether municipalities were adjacent to each other using the map of Japan. Therefore, only municipalities adjacent to each other were used in the subsequent analysis. Further, a conditional autoregressive model by spatialreg (https://cran.r-project.org/web/packages/spatialreg/spatialreg.pdf (accessed on 30 November 2021)) was used in the analysis, and the spatial correlation among municipalities was taken into account in the regression analysis. In a conditional autoregressive model, the correlation between error terms of adjacent municipalities was taken into account [28]. In addition, the SMR was log-transformed in the analysis because its distribution was right-skewed. The outcome and explanatory variables were both scaled, and a standardized partial regression coefficient (SPRC), 95% confidence interval (CI), and p-value were calculated for each explanatory variable by a multivariate regression analysis. A *p*-value < 0.05 was considered statistically significant.

We checked the multi-collinearity of the explanatory variables based on the variance inflation factor (VIF). If multi-collinearity exists, the VIF of an explanatory variable increases. Although there are no standard criteria for VIF, a value above 5 or 10 is often used as criteria for multi-collinearity [29]. Package car (https://cran.r-project.org/web/packages/car/car.pdf (accessed on 30 November 2021)) was used for calculating VIF. The interaction terms of the explanatory variables were not included in the analysis, because we conducted an exploratory analysis using multiple kinds of variables. If we include interaction terms for them, the number of the explanatory variables will exceed 100.

All statistical analyses were conducted using R3.6.3 (https://www.R-project.org/ (accessed on 30 November 2021)).

## 3. Results

**Figure 1:**
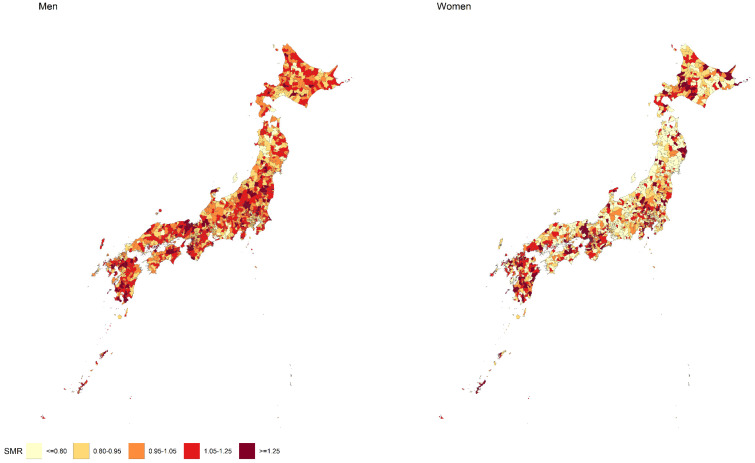
Geographic differences in standardized mortality ratio (SMR) of chronic obstructive pulmonary disease (COPD) for men and women in Japan.

The geographical differences in SMR of COPD for men and women in Japan are shown in Figure 1, and the SMRs of all the municipalities in Japan are shown. Geographical differences in the SMR were different between men and women. Municipalities with SMRs > 1.05 were observed more often in men, whereas many municipalities with SMR < 0.80 tended to be observed in women. In women, the SMRs tended to be low in Tohoku areas, whereas there were many municipalities with high SMR in Kyushu and Kinki areas. Skewness of SMR in men was 0.60, whereas that in women was 2.84. Therefore, the distribution of SMR in women is much right-skewed than that in men.

The top 10 municipalities with the highest SMR of COPD for men and women in Japan are shown in Table 1. There were some overlaps in the municipalities among men and women, and it was revealed that the SMR of Tanba city was particularly high in women.

There were 1741 municipalities in the analyzed periods; 1687 municipalities were used in the subsequent analysis after excluding municipalities not adjacent with another and municipalities with no sufficient census data in the periods.

The basic characteristics of the analyzed data are observed in Table 2. Twelve types of explanatory variables were used in the analysis. It can be confirmed that some characteristics, such as population and number of hospital beds, largely vary among municipalities. Additionally, COPD mortality in men was five-times larger than that in women. As a result of evaluating VIF, the largest value was 3.44 of the proportion of divorced persons, and the values of the other variables were in the range of 1 to 3. Therefore, it is considered that a large multi-collinearity did not exist among the explanatory variables.

The results of the multivariate regression analysis for identifying predictors of COPD mortality can be seen in Table 3. The total population and taxable income per capita were negatively associated with the SMR in men. In women, population density, the proportion of fatherless households, and the number of clinics per capita were positively associated with the SMR, whereas taxable income per capita was negatively associated with the SMR.

## 4. Discussion

We identified geographical differences in COPD mortality in Japan and identified factors associated with the SMR. We discuss about possible reason for the association between COPD mortality and the identified factors in below.

Although there were some similarities among municipalities with the highest SMR in men and women, the geographic distribution of the SMR differed by sex. This can be explained by the fact that SMR predictors are different for the sexes, for example, it was shown that the proportion of fatherless households and population density are SMR predictors in women but not in men. Another reason is that the COPD mortality rate largely differed by sex. There were many municipalities with low COPD mortality in women, leading to the low SMR in women in those municipalities. One possible factor for this difference observed between sexes is smoking; smoking is a major cause of COPD incidence in Japan [4]. Although it was shown that susceptibility to lung-damaging effects of smoking in women is higher than in men [30,31], smoking prevalence in Japan is much higher in men than in women [32]. Additionally, the SMRs of top-ranked municipalities for women were much higher than men. In women, there were many municipalities with low COPD mortality, and there were few municipalities with high COPD mortality. As a result, the distribution of the SMR was much right-skewed among women compared with that among men, and it is considered to be related to the high SMR values of the top-ranked municipalities in women.

The total population was negatively associated with COPD mortality in men. Urbanization is often associated with increased COPD risk in other countries because urbanization often worsens air pollution [33,34]. In contrast, an increased COPD mortality rate in rural areas compared with urban areas has been shown in the United States [35]. The relationship between COPD mortality (or prevalence) and urbanization is considered different depending on the country. Urban–rural disparities in medical care, which patients with COPD can obtain, have been reported in other countries [36,37]. Geographical differences in home oxygen use have been identified in another county [38], and there is a possibility that in Japan, a patient with COPD cannot fully obtain home oxygen therapy in rural areas. Urban and rural differences in spirometry use have also been identified in another country [39].

On the other hand, there was a positive association between COPD mortality and population density in women, and smoking prevalence is one possible reason since the geographic differences in smoking prevalence differ by sex in Japan. Smoking is a major risk factor for COPD mortality in Japan [9,40]. Smoking prevalence in urban areas is known to be high compared with non-urban areas among older Japanese women [32], and it is considered to have affected the results for women. In Japan, regional urbanization has been associated with smoking behavior in women [41]. In contrast, smoking prevalence in older Japanese men in urban areas is not higher compared with non-urban areas [32], and it may be linked to the negative association between population and COPD mortality in men. Smoking prevalence is believed to be directly related to COPD prevalence rather than COPD mortality, and investigating regional differences in COPD prevalence will be of significance in the future. Another possible reason for the positive association between COPD mortality and population density in women is access to medical facilities. It is possible that a patient with COPD is more likely to be diagnosed with COPD in urban areas due to easy access to medical facilities.

The taxable income per capita was negatively associated with COPD mortality in men, and the proportion of fatherless households was positively associated with COPD mortality in women. Fatherless households are associated with low income in Japan [42] and are an indicator of the prevalence of women in poverty. Smoking prevalence is higher among persons with a low socioeconomic status in Japan [43], and a negative association between income and smoking prevalence has been shown [44]. Income is also related to consultation behavior for medical care. Non-attendance of health check-ups increases as income decreases in Japan [41], and elderly persons with lower incomes attend fewer physician consultations [45]. Therefore, COPD detection may be delayed among persons with a low income or low socioeconomic status.

The number of clinics per capita was also positively associated with the SMR for women. One possible reason for the association between the number of medical clinics per capita mortality is that a person tends to be diagnosed with COPD in regions with a high number of medical institutions. It is known that a large number of patients with COPD are not diagnosed with COPD in Japan [13], and a patient may tend not to be diagnosed with COPD in regions where the number of medical institutions is few in the neighborhood. The cause of death in those patients with no diagnosis is not necessarily COPD unless the lungs are autopsied. In addition, patients with COPD may change their residence to regions where they can easily access medical institutions.

It was found that socioeconomic status is related to geographical differences in COPD mortality in Japan both among men and women. Smoking is considered a major factor, and lowering smoking prevalence in persons with a low income is necessary to ease regional differences. Raising the price of cigarettes has been proposed to ease the disparity in smoking [43], and it will possibly contribute to a decrease in the regional differences in COPD mortality. In addition, detecting COPD early in regions where medical institutions are few might be a future challenge. A survey investigating regional differences in medical care and diagnostic tools, such as home oxygen therapy or spirometry use, may also be needed to understand this study’s results. Furthermore, public awareness of COPD is still low in Japan [4], and increased public awareness will contribute to early diagnosis. Furthermore, more diagnostic testing opportunities should be offered for residents in regions with a higher SMR to detect undiagnosed patients with COPD.

It was found that the number of clinics per capita was positively associated with COPD mortality. Therefore, using the medical characteristics will be meaningful also for studies in other countries. Additionally, the results suggested underdiagnosis of COPD in regions with inferior medical facilities, and mortality data may be affected by such regional underdiagnosis also in other countries. Investigating regional differences in the prevalence of undiagnosed COPD will also be of value in addressing this issue. Mass screening or telemedicine is a timely way for detecting COPD in patients in regions with inferior medical facilities. Furthermore, the results of this study suggest that the early detection of COPD in patients in developing countries, where medical equipment is limited and income level is low, is a future task for global health.

There are some limitations to this study. COPD mortality tends to be underreported globally [46], and it is considered that actual COPD mortality is larger than that reported in the Vital Statistics. Second, this is an ecological study, and an ecological fallacy might exist for the results of the identified factors. An epidemiological study investigating COPD mortality and identifying predictors using individual data is needed to verify the results of this study. Third, other unused predictors associated with COPD mortality might exist. For example, the concentration of air pollutants, such as particulate matter 2.5, is possibly related to COPD or chronic respiratory mortality [33,47,48], and municipal-specific data for air pollutants could not be obtained.

## 5. Conclusions

We identified geographical differences in COPD mortality in Japan using municipal-specific data and identified factors associated with COPD mortality. As a result, geographical differences in the SMR were different between men and women, and municipalities with low SMR tended to be observed more often in women. Spatial regression analysis identified that the total population and taxable income per capita were negatively associated with the SMR in men. In women, population density, the proportion of fatherless households, and the number of clinics per capita were positively associated with the SMR, whereas taxable income per capita was negatively associated with the SMR. There were some differences in regional characteristics associated with COPD mortality by sex. A survey investigating the regional differences in medical care, diagnostic tools for COPD, and the effects of underdiagnosed COPD will be significant in the future.

## Figures and Tables

**Table 1:** The top 10 municipalities with the highest SMR of COPD for men and women in Japan.

Rank	Men		Women	
	Municipality Name (Prefecture Name)	SMR	Municipality Name (Prefecture Name)	SMR
1	Mima city (Tokushima)	1.86	Tanba city (Hyogo)	5.22
2	Inabe city (Mie)	1.77	Ichikikushikino city (Kagoshima)	2.95
3	Uruma city (Okinawa)	1.74	Okinawa city (Okinawa)	2.74
4	Tanba city (Hyogo)	1.74	Echizen city (Fukui)	2.68
5	Yame city (Fukuoka)	1.65	Itoman city (Okinawa)	2.58
6	Nagashima town (Kagoshima)	1.64	Mima city (Tokushima)	2.58
7	Numata city (Gunma)	1.61	Uruma city (Okinawa)	2.55
8	Isa city (Kagoshima)	1.61	Omuta city (Fukuoka)	2.50
9	Fukuchiyama city (Kyoto)	1.60	Hachinohe city (Aomori)	2.41
10	Kinogawa city (Wakayama)	1.56	Kamaishi city (Iwate)	2.31

**Table 2:** Basic characteristics of the analyzed data.

Municipal Characteristics	Median (Interquartile Range)
	(N = 1687)
Total population	26,752 (9110–67,438)
Population density (person per hectare)	2.1 (0.6–8.2)
Proportion of single households (%)	27.3 (22.8–32.5)
Proportion of fatherless households (%)	1.4 (1.1–1.7)
Proportion of divorced persons (%)	5.0 (4.3–5.8)
Proportion of unemployed persons (%)	3.9 (3.3–4.7)
Proportion of blue-collar workers (%)	7.3 (6.5–8.4)
Taxable income per capita (1000 yen)	1103.3 (931.3–1293.0)
Number of hospital beds ^1^	1026.7 (430.4–1694.5)
Number of clinics ^1^	68.4 (54.1–84.2)
Number of hospitals ^1^	6.0 (2.1–10.1)
Number of physicians ^1^	128.8 (74.4–194.1)
Male COPD mortality rate ^1^	26.4 (18.6–37.3)
Female COPD mortality rate ^1^	4.6 (2.5–7.6)

^1^ Number per 100,000 persons.

**Table 3:** Results of the multivariate regression analysis for identifying predictors of COPD mortality.

Explanatory Variables	Men		Women	
	SPRC (95% CI)	*p*-Value	SPRC (95% CI)	*p*-Value
Total population	−0.060 (−0.112, −0.009)	0.021	0.018 (−0.033, 0.070)	0.488
Population density	−0.008 (−0.078, 0.061)	0.812	0.148 (0.079, 0.217)	0.000
Proportion of single households (%)	−0.051 (−0.118, 0.017)	0.142	0.043 (−0.025, 0.110)	0.215
Proportion of fatherless households (%)	0.008 (−0.063, 0.079)	0.824	0.137 (0.067, 0.208)	0.000
Proportion of divorced persons (%)	0.049 (−0.036, 0.134)	0.258	0.043 (−0.043, 0.128)	0.327
Proportion of unemployed persons (%)	−0.024 (−0.083, 0.036)	0.433	0.020 (−0.039, 0.080)	0.503
Proportion of blue-collar workers (%)	0.000 (−0.053, 0.053)	0.998	0.004 (−0.049, 0.058)	0.873
Taxable income per capita	−0.215 (−0.285, −0.144)	0.000	−0.129 (−0.200, −0.059)	0.000
Number of hospital beds ^1^	−0.020 (−0.095, 0.056)	0.612	0.050 (−0.026, 0.126)	0.200
Number of clinics ^1^	0.046 (−0.008, 0.099)	0.092	0.086 (0.032, 0.139)	0.002
Number of hospitals ^1^	0.065 (−0.003, 0.134)	0.062	0.040 (−0.028, 0.109)	0.250
Number of physicians ^1^	0.014 (−0.047, 0.074)	0.657	−0.054 (−0.115, 0.006)	0.079

SPRC: Standardized partial regression coefficient; CI: confidence interval; ^1^ Number per 100,000 persons.

## Data Availability

All the data used in this study are publicly available. The source of the data used in this study are written in the References.
